# Organic Fertilizer Combined with Microbial Inoculant Alleviates Saline–Alkali Stress in Wheat: A Multi-Tissue Analysis

**DOI:** 10.3390/biology15171467

**Published:** 2026-08-29

**Authors:** Min Li, Hongxia Liu, Na Wang, Junlong Wang, Mengyao Duan, Mei Liao, Xingang Zhou, Pengfei Chu, Xuewen Hua, Junkang Sui

**Affiliations:** 1 College of Agriculture and Biology, Liaocheng University, Liaocheng 252000, China; 2 College of Horticulture, Northeast Agricultural University, Harbin 150006, China

**Keywords:** wheat rhizosphere, soil amelioration, microbial community reassembly, enzymatic antioxidant system, oxidative homeostasis

## Abstract

Soil salinization constrains global wheat production, but how organic–microbial amendments alleviate saline–alkali stress across plant tissues remains poorly characterized. In a single-season field trial, we compared three treatments—a non-saline control (CK), a saline–alkali control (SCK), and an organic fertilizer combined with a microbial inoculant applied to saline–alkali soil (SDM)—to evaluate effects on soil properties, the rhizosphere microbiome structure, leaf antioxidant defense, and grain quality. SDM improved soil nutrient availability: available phosphorus and potassium increased by 96.0% and 51.8%, respectively, relative to SCK, and rhizosphere microbiome shifts favored copiotrophic taxa (*Flavobacterium*, *Devosia*, *Mortierella*) over halotolerant genera (*Arthrobacter*, *Filobasidium*). In leaves, SDM induced a controlled antioxidant activation—peroxidase (POD) functioned as a key mediator of oxidative homeostasis, with activities exceeding CK yet remaining below extreme SCK levels—producing the lowest malondialdehyde content (−48.4% vs. SCK). At moderate concentrations, reactive oxygen species participated in developmental signaling rather than causing pathological damage, enabling grain soluble sugars and total starch to be restored to CK levels. These results reveal a coordinated soil–microbiome–plant response in which organic–microbial amendment enhances nutrient cycling, redirects community assembly toward nutrient-cycling functional guilds, and achieves oxidative homeostasis and grain quality restoration. Our findings offer a conceptual basis for the microbiome-informed management of saline–alkali wheat production.

## Simple Summary

Saline–alkaline soils reduce wheat yields by 20–50%. This study tested whether organic fertilizer combined with salt-tolerant beneficial microbes could alleviate this stress. Compared with untreated saline–alkali soil, the treatment nearly doubled soil phosphorus, shifted root microbes from stress-tolerators toward nutrient recyclers, and enabled controlled leaf antioxidant activation with minimal cell damage. Grain sugar and starch recovered to healthy-soil levels. These linked soil–microbe–leaf–grain improvements suggest that enhancing soil biology can alleviate saline–alkaline stress throughout wheat plants, offering a practical path for reclaiming degraded farmland.

## 1. Introduction

Soil salinization affects an estimated 1.1 billion hectares of land globally and is among the most persistent threats to agricultural sustainability. Approximately 20% of irrigated cropland experience some degree of salt-induced degradation, with the problem concentrated in arid and semi-arid regions—including major wheat-producing areas of China, South Asia, and Australia—where high evapotranspiration and limited freshwater for leaching drive progressive salt accumulation in the root zone [1,2]. Beyond imposing direct osmotic and ionic stress on plants, salinization fundamentally alters soil microbial communities: it suppresses microbial biomass and enzymatic activity while favoring halotolerant taxa over decomposer populations, with cascading consequences for nutrient cycling and soil fertility [3]. Wheat (*Triticum aestivum* L.), a staple cereal that feeds over 35% of the global population, is classified as a moderately salt-sensitive glycophyte, and its grain yield can decline by 20–50% under moderate to severe saline–alkali stress [4]. This yield penalty, set against the projected need to increase cereal production by 60% by 2050, positions saline–alkali wheat production as a pressing food security challenge [5].

Saline–alkali stress imposes a multi-tiered physiological burden on wheat. At the root level, elevated Na^+^ concentrations and high pH disrupt the ionic balance of the soil solution, impairing the uptake of essential nutrients—particularly K^+^, phosphorus, and nitrogen—while osmotic stress reduces water availability [6]. At the cellular level, ionic toxicity and osmotic imbalance trigger the overproduction of reactive oxygen species (ROS), including superoxide anion (O_2_^−^), hydrogen peroxide (H_2_O_2_), and hydroxyl radicals. When scavenging capacity is exceeded, these ROS cause lipid peroxidation, protein oxidation, and chlorophyll degradation. Plants counter oxidative challenge through a coordinated antioxidant defense system: enzymatic components include superoxide dismutase (SOD), peroxidase (POD), and catalase (CAT), while non-enzymatic players encompass ascorbate, glutathione, and osmoprotectants such as proline and soluble sugars [7]. Sustained activation of this defense system under chronic stress, however, diverts carbon and nitrogen resources away from growth and grain filling [8,9]. At the regulatory level, the NRF2/ARE signaling pathway and heat shock transcription factors serve as key hubs that integrate redox sensing with the transcriptional reprogramming of antioxidant and cytoprotective genes [10,11].

Current strategies for saline–alkali soil amelioration include chemical, physical, biological, and genetic approaches. Chemical amendments such as gypsum and sulfuric acid effectively displace exchangeable Na^+^, but their high material costs and the risk of secondary soil acidification limit widespread application [12]. Salt-tolerant wheat varieties developed through conventional breeding and molecular approaches offer genetic solutions, though they typically involve trade-offs between stress tolerance and yield potential. Among biological strategies, organic fertilizer application has gained attention as a cost-effective and environmentally sustainable option: it improves soil structure, enhances cation exchange capacity, and supplies plant-available nutrients [13]. Under saline–alkali conditions, however, high pH and osmotic stress constrain microbial metabolic activity, slowing the mineralization of organic amendments and limiting short-term nutrient release needed for rapid crop establishment [14]. This bottleneck has motivated interest in combined organic–microbial strategies, in which microbial inoculants containing plant growth-promoting rhizobacteria (PGPR) and beneficial fungi are co-applied with organic fertilizers. The co-application is intended to accelerate organic matter decomposition, enhance nutrient solubilization, and supply direct plant-growth-promoting functions [15,16]. The SDM treatment examined here—an organic fertilizer base supplemented with a composite microbial inoculant—represents one such integrated approach.

The rhizosphere microbiome functions as a critical intermediary between soil amendments and plant performance. It has been conceptualized as a plant’s “second genome,” with community composition and functional capacity directly shaping nutrient acquisition, pathogen defense, and abiotic stress tolerance [17]. Under saline–alkali conditions, strong environmental filtering—driven by high pH, elevated electrical conductivity (EC), and Na^+^ toxicity—selectively enriches halotolerant and oligotrophic taxa at the expense of community diversity [18]. Organic matter inputs can partially counteract this filtering: labile carbon substrates preferentially support copiotrophic groups with higher metabolic rates and greater capacities for extracellular enzyme production, shifting community composition from slow-growing, stress-adapted oligotrophs toward faster-growing, nutrient-cycling functional guilds [19]. Biochar and organic amendments also confer physical and chemical benefits beyond microbial modulation—improving soil pore structure, enhancing water retention, and reducing CO_2_ emissions in wheat–rice rotation systems [20]. Critically, most studies to date have examined soil physicochemical properties or microbial community responses in isolation. Whether soil and microbiome improvements induced by organic–microbial amendments translate into coordinated benefits across plant tissues—particularly at the leaf (antioxidant defense) and grain (quality) levels—remains largely unexplored.

Here, we used a three-group experimental design—a non-saline control (CK), a saline–alkali control (SCK), and SDM treatment (organic fertilizer + microbial inoculant under saline–alkali conditions)—to systematically characterize the cascading effects of organic–microbial amendment at four interconnected biological levels: (1) soil physicochemical properties; (2) rhizosphere microbial community structure (α- and β-diversity, phylum- and genus-level composition, differential OTU analysis, and Mantel-based environmental driver identification for both bacteria and fungi); (3) leaf antioxidant enzyme activities and oxidative stress markers; and (4) grain antioxidant profiles and quality parameters (crude protein, total starch, soluble sugars). We tested three hypotheses—that SDM would (i) improve soil nutrient availability and reduce rhizosphere ionic stress; (ii) redirect the rhizosphere microbiome from a saline–alkali-associated, oligotroph-dominated state toward a copiotrophic, nutrient-cycling community; or (iii) produce a coordinated multi-tissue plant response in which enhanced nutrient supply and microbially mediated stress buffering at the root level would translate into controlled leaf antioxidant activation and partial restoration of grain quality.

## 2. Materials and Methods

### 2.1. Experimental Site and Design

The field experiment was conducted at the experimental station of Liaocheng Chuangju Fengwanjiang Agricultural Technology Development Co., Ltd. (115.77° E, 36.53° N), Dongchangfu District, Liaocheng, Shandong Province, China. This region experiences a warm temperate monsoon climate with a mean annual temperature of 12.8–13.4 °C, an annual precipitation of 567.7–637.3 mm, and approximately 200 frost-free days per year. The soil at the experimental site is classified as fluvo-aquic soil with saline–alkali characteristics. CK and SCK plots were established on adjacent fields (approximately 50 m apart) sharing the same fluvo-aquic soil parent material and long-term cultivation history; the salinity difference reflects differential historical irrigation and drainage rather than an experimentally imposed salt treatment. Three treatment groups were established in a randomized block design with three replicate plots (20 m × 20 m) per treatment: (1) CK—non-saline control soil with no amendment; (2) SCK—saline–alkali soil with no amendment; and (3) SDM—saline–alkali soil treated with organic fertilizer (decomposed sheep manure, 15 t/ha) combined with a composite microbial inoculant. The composite microbial inoculant consisted of three individually prepared components: *Bacillus subtilis* (≥2 × 10^8^ CFU/g), produced by liquid fermentation on soybean meal and corn flour, absorbed onto a sterilized carrier after concentration; *Bacillus licheniformis* (≥1 × 10^8^ CFU/g), prepared similarly by liquid fermentation and carrier absorption; and *Trichoderma harzianum* (≥5 × 10^7^ CFU/g), cultured by solid-state fermentation on a wheat-bran substrate. The three components were blended to form the final formulated product. The inoculant was applied at 75 kg/ha by mixing thoroughly with the organic fertilizer and incorporating into the top 20 cm of soil one week before wheat sowing (October 2024). All three strains were isolated from the rhizosphere of wheat grown under saline–alkali field conditions at the experimental site and were selected based on in vitro screening for salt tolerance and multiple plant growth-promoting traits (including ACC deaminase activity, IAA production, siderophore production, and phosphate solubilization). Winter wheat (*Triticum aestivum* L. cv. Jimai 22) was sown in October 2024 and harvested in June 2025. Standard field management practices including irrigation and weed control were applied uniformly across all treatments. We note that the present study is a single-site, single-season field trial; therefore, the findings should be interpreted as site- and season-specific until validated across broader environmental conditions.

### 2.2. Soil Sampling and Physicochemical Analysis

At the grain-filling stage (late May 2025), rhizosphere soil samples were collected from five randomly selected wheat plants per plot at a depth of 15–20 cm. After removing bulk soil by gentle shaking, the soil tightly adhering to the root surface was collected as rhizosphere soil using a sterile brush. Samples from each plot were pooled, homogenized, and divided into two portions: one was air-dried and sieved (2 mm) for physicochemical analyses, and the other was immediately frozen in liquid nitrogen and stored at −80 °C for DNA extraction. Soil physicochemical properties were determined following standard protocols. Total nitrogen (TN) was measured by the Kjeldahl digestion method; total phosphorus (TP) was determined by molybdenum–antimony colorimetry after H_2_SO_4_-H_2_O_2_ digestion; and total potassium (TK) was quantified by flame photometry after NaOH fusion. Alkaline hydrolyzable nitrogen (AN) was measured by the alkaline hydrolysis diffusion method; available phosphorus (AP) was extracted with 0.5 M NaHCO_3_ and determined by the molybdenum blue method; and available potassium (AK) was extracted with 1 M NH_4_OAc and measured by flame photometry. Soil organic matter (OM) was determined by the potassium dichromate oxidation–external heating method. Soil pH was measured in a 1:2.5 (w/v) soil–water suspension using a glass electrode pH meter. Electrical conductivity (EC) was measured in a 1:5 soil–water extract using a conductivity meter. Soil moisture content was determined gravimetrically by oven-drying at 105 °C to constant weight.

### 2.3. Leaf and Grain Sampling and Biochemical Assays

At the grain-filling stage, flag leaves were collected from five plants per plot, immediately frozen in liquid nitrogen, and stored at −80 °C. At maturity, wheat spikes were harvested and threshed manually, and the grains were ground to a fine powder for biochemical analyses. All biochemical assays were performed by Wuhan Pronets Biotechnology Co., Ltd. (Wuhan, China) using validated protocols. Superoxide dismutase (SOD) activity was determined by the nitroblue tetrazolium (NBT) photoreduction method, with one unit defined as the enzyme amount causing 50% inhibition of NBT reduction at 560 nm. Peroxidase (POD) activity was measured by the guaiacol colorimetric method at 470 nm. Catalase (CAT) activity was assayed by monitoring the decomposition of H_2_O_2_ at 240 nm. Malondialdehyde (MDA) content was determined by the thiobarbituric acid (TBA) method at 532 nm. Hydrogen peroxide (H_2_O_2_) content was measured by the titanium sulfate colorimetric method at 415 nm. Superoxide anion (O_2_^−^) content was determined by the hydroxylamine oxidation method at 530 nm. Soluble sugar content was measured by the anthrone–sulfuric acid colorimetric method at 620 nm. Soluble protein content was determined by the Coomassie Brilliant Blue G-250 dye-binding method at 595 nm. Proline content was measured by the ninhydrin colorimetric method at 520 nm. Chlorophyll content was extracted with 95% ethanol and determined spectrophotometrically at 665 and 649 nm. Grain crude protein content was calculated by multiplying the Kjeldahl nitrogen content by a factor of 5.70. The total starch content in grains was determined by the anthrone-sulfuric acid method. All assays were performed with three biological replicates, each measured in technical triplicate.

### 2.4. DNA Extraction, PCR Amplification, and Illumina Sequencing

Total microbial genomic DNA was extracted from 0.5 g of rhizosphere soil using the E.Z.N.A.^®^ Soil DNA Kit (Omega Bio-tek, Norcross, GA, USA) according to the manufacturer’s instructions. DNA concentration and purity were assessed using a NanoDrop 2000 spectrophotometer (Thermo Fisher Scientific, Wilmington, DE, USA), and integrity was verified by 1% agarose gel electrophoresis. The V3–V4 hypervariable region of the bacterial 16S rRNA gene was amplified using primer pairs 338F (5′-ACTCCTACGGGAGGCAGCAG-3′) and 806R (5′-GGACTACHVGGGTWTCTAAT-3′) [21]. The ITS1 region of the fungal rRNA gene was amplified using primer pairs ITS1F (5′-CTTGGTCATTTAGAGGAAGTAA-3′) and ITS2R (5′-GCTGCGTTCTTCATCGATGC-3′) [22]. PCR amplification was performed on an ABI GeneAmp^®^ 9700 thermocycler (Applied Biosystems, Foster City, CA, USA) in a 20 μL reaction mixture containing 4 μL of 5× FastPfu buffer, 2 μL of 2.5 mM dNTPs, 0.8 μL of each primer (5 μM), 0.4 μL of FastPfu polymerase (TransGen Biotech, Beijing, China), 10 ng of template DNA, and ddH_2_O to volume. Amplification conditions were: initial denaturation at 95 °C for 3 min; 27 cycles of 95 °C for 30 s, 55 °C for 30 s, and 72 °C for 45 s; and a final extension at 72 °C for 10 min. Triplicate amplifications were performed for each sample. PCR products were purified using the AxyPrep DNA Gel Extraction Kit (Axygen Biosciences, Union City, CA, USA) and quantified with a Quantus™ Fluorometer (Promega, Madison, WI, USA). Purified amplicons were pooled in equimolar concentrations and paired-end sequenced (2 × 300 bp) on the Illumina MiSeq PE300 platform (Illumina, San Diego, CA, USA) by Majorbio Bio-Pharm Technology Co., Ltd. (Shanghai, China).

### 2.5. Bioinformatics and Statistical Analysis

Raw FASTQ files were demultiplexed, quality filtered using fastp (v0.19.6) [23], and merged with FLASH (v1.2.7) [24]. Sequences with an average quality score <20 over a 50 bp sliding window were truncated, and reads shorter than 50 bp or containing ambiguous bases were discarded. Operational taxonomic units (OTUs) were clustered at 97% sequence similarity using UPARSE (v7.1) [25], and chimeric sequences were identified and removed using UCHIME. The taxonomic classification of representative sequences was performed against the SILVA 138 database for bacteria and the UNITE 8.0 database for fungi using the RDP Classifier with a confidence threshold of 0.7. To minimize the effects of uneven sequencing depth, all samples were rarefied to the minimum sequencing depth prior to downstream analyses. After quality filtering and chimera removal, the minimum rarefaction depth was 62,159 reads per sample for bacteria (total 617,483 reads across 9 samples) and 99,062 reads per sample for fungi (total 961,794 reads). Good’s coverage exceeded 0.99 for all libraries. Per-sample sequencing and rarefaction statistics are provided in Table S4.

Alpha diversity indices (Sobs, Shannon, Simpson, ACE, Chao1, and coverage) were calculated using Mothur (v1.30.1). Beta diversity was assessed by principal coordinate analysis (PCoA) based on Bray–Curtis distance matrices, and statistical significance of community separation was evaluated by PERMANOVA with 999 permutations. A differential OTU analysis between treatment pairs was performed using Welch’s *t*-test with a significance threshold of |log2 fold change| > 1 and *p* < 0.05. Environmental drivers of microbial community structure were identified by a Mantel test analysis (99 permutations) relating Bray–Curtis community distance matrices to Euclidean distance matrices of environmental variables.

Soil, leaf, and grain biochemical data were analyzed using Welch’s *t*-test for pairwise comparisons among CK, SDM, and SCK groups. Significance levels were indicated as: ns (not significant, *p* ≥ 0.05), * *p* < 0.05, ** *p* < 0.01, and *** *p* < 0.001. All statistical analyses were performed in Python (v3.13) using the SciPy (v1.18) and Pandas (v2.5) libraries. Boxplots and heatmaps were generated using Matplotlib (v3.11) and saved at 300 dpi resolution. The bioinformatic workflow was partially executed on the Majorbio Cloud Platform (https://cloud.majorbio.com accessed on 26 June 2026). For OTU-level differential abundance analyses, a more conservative threshold of *p* < 0.01 (Welch’s *t*-test) combined with |log_2_ fold change| > 1 was applied. We adopted this stricter raw threshold rather than a false discovery rate (FDR) correction because, with *n* = 3 replicates and thousands of OTUs tested simultaneously, the Benjamini–Hochberg correction was excessively conservative (retaining only 25 bacterial OTUs at *q* < 0.05), which would have discarded biologically meaningful signals. The FDR analysis was nevertheless performed as a sensitivity check, and its outcome was consistent with the *p* < 0.01 result. OTU-level differential lists should therefore be regarded as exploratory.

## 3. Results

### 3.1. Soil Physicochemical Properties

**Figure 1:**
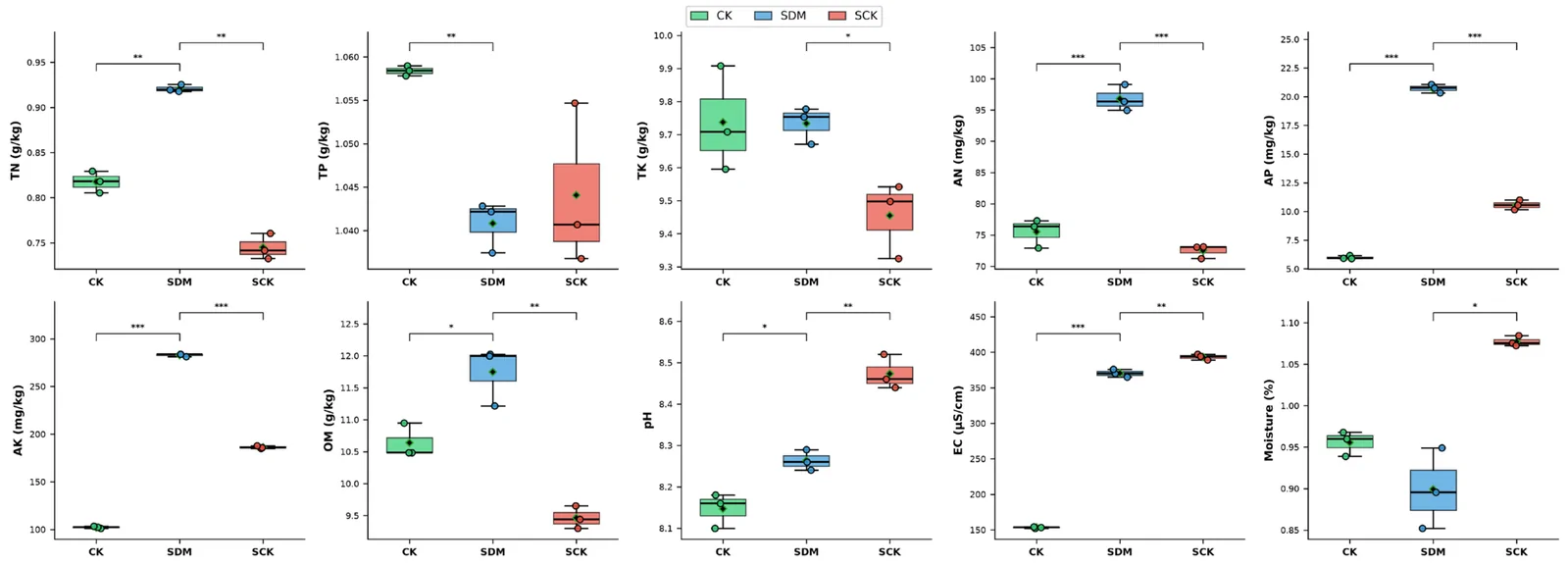
Soil physicochemical properties of CK (non-saline control), SDM (organic fertilizer + microbial inoculant), and SCK (saline–alkali control) treatments. Boxplots show the median (center line), interquartile range (box), 1.5 × IQR (whiskers), and individual replicate values (jittered points, *n* = 3 per treatment). Mean values are indicated by black diamonds. Asterisks denote significant differences between SDM and CK or SDM and SCK (Welch’s *t*-test: ns, *p* ≥ 0.05; * *p* < 0.05; ** *p* < 0.01; *** *p* < 0.001).

Post-harvest soil measurements confirmed that SCK plots maintained saline–alkali characteristics throughout the experiment, with a mean EC of 393 μS/cm and pH of 8.47 (Table S1). These values are consistent with the Chinese saline–alkali soil classification threshold (EC > 200 μS/cm, pH > 8.0) and significantly exceed the non-saline CK levels (EC 153 μS/cm, pH 8.15). SDM treatment significantly improved soil nutrient availability relative to both CK and SCK (Table S1; Figure 1). Total nitrogen (TN) in SDM soil reached 0.92 ± 0.00 g/kg, exceeding SCK (0.75 ± 0.01 g/kg) by 23.6% (** *p* < 0.01) and CK (0.82 ± 0.01 g/kg) by 12.6% (** *p* < 0.01). Alkaline hydrolyzable nitrogen (AN) showed a similarly strong response, reaching 96.80 ± 2.11 mg/kg in SDM—33.5% above SCK (72.48 ± 1.06 mg/kg, *** *p* < 0.001) and 28.2% above CK (75.52 ± 2.30 mg/kg, *** *p* < 0.001).

The largest relative improvement was in available phosphorus (AP), which reached 20.72 ± 0.37 mg/kg under SDM—a 245.4% increase over CK (6.00 ± 0.14 mg/kg, *** *p* < 0.001) and 96.0% increase over SCK (10.57 ± 0.42 mg/kg, *** *p* < 0.001). Available potassium (AK) in SDM (282.89 ± 1.42 mg/kg) was 51.8% higher than SCK (186.30 ± 1.46 mg/kg, *** *p* < 0.001) and 176.6% higher than CK (102.28 ± 0.96 mg/kg, *** *p* < 0.001). Total phosphorus (1.04 ± 0.00 g/kg) and total potassium (9.73 ± 0.06 g/kg) were comparable to SCK, suggesting that SDM primarily enhanced nutrient availability rather than expanding total pools. Organic matter (OM) content reached 11.75 ± 0.46 g/kg in SDM, +24.1% versus SCK (9.47 ± 0.18 g/kg, ** *p* < 0.01) and +10.4% versus CK (10.64 ± 0.27 g/kg, * *p* < 0.05).

Soil pH in SDM (8.26 ± 0.03) was lower than SCK (8.47 ± 0.04, ** *p* < 0.01, −2.5%) but remained slightly above CK (8.15 ± 0.04, * *p* < 0.05). Electrical conductivity (EC) decreased from 393.33 ± 4.04 μS/cm in SCK to 370.33 ± 5.51 μS/cm in SDM (** *p* < 0.01, −5.8%), though it stayed substantially elevated relative to CK (153.37 ± 0.97 μS/cm, *** *p* < 0.001). Soil moisture under SDM (0.90 ± 0.05) was lower than SCK (1.08 ± 0.01, * *p* < 0.05, −16.6%) and comparable to CK (0.96 ± 0.01, ns).

### 3.2. Rhizosphere Microbial Community Structure

#### 3.2.1. Alpha Diversity

**Figure 2:**
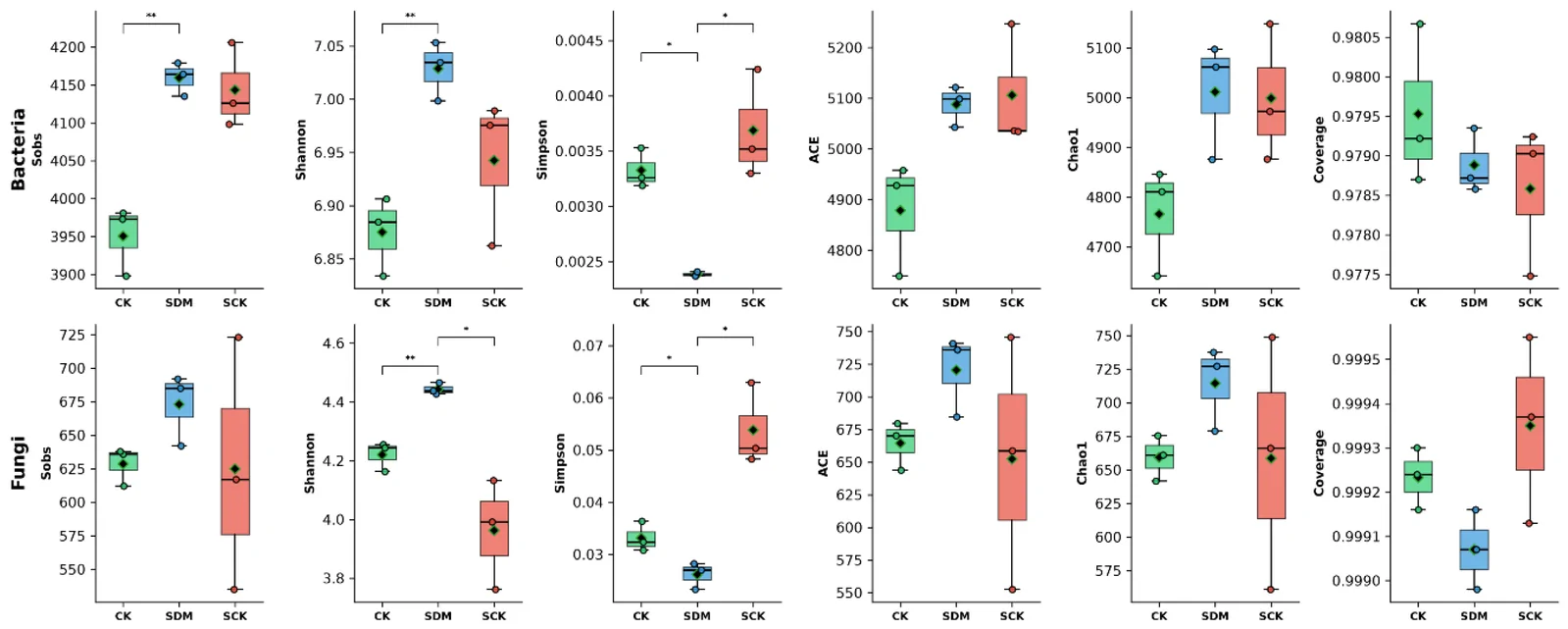
Alpha diversity indices of rhizosphere bacterial and fungal communities across CK, SDM, and SCK treatments. Upper row: bacteria; lower row: fungi. Six indices are displayed horizontally: observed species (Sobs), Shannon, Simpson, ACE, Chao1, and coverage (*n* = 3 per treatment). Box elements and statistical annotations are as in Figure 1. Good’s coverage values exceeded 0.99 for all bacterial and fungal libraries, indicating that sequencing depth was sufficient to capture the majority of microbial diversity (Table S4).

SDM treatment significantly enhanced bacterial community diversity. The bacterial Shannon index in SDM (7.03 ± 0.03) exceeded CK (6.88 ± 0.04, * *p* < 0.05) and trended higher than SCK (6.94 ± 0.07, ns). Fungal Shannon diversity followed a parallel pattern: SDM (4.44 ± 0.02) was higher than both CK (4.22 ± 0.05, * *p* < 0.05) and SCK (3.96 ± 0.19, * *p* < 0.05). Sobs, Chao1, and ACE indices confirmed consistent trends for both bacterial and fungal communities. Simpson and coverage indices did not differ significantly among treatments, indicating that α-diversity differences reflected rare taxa rather than dominant species turnover (Figure 2).

#### 3.2.2. Beta Diversity

**Figure 3:**
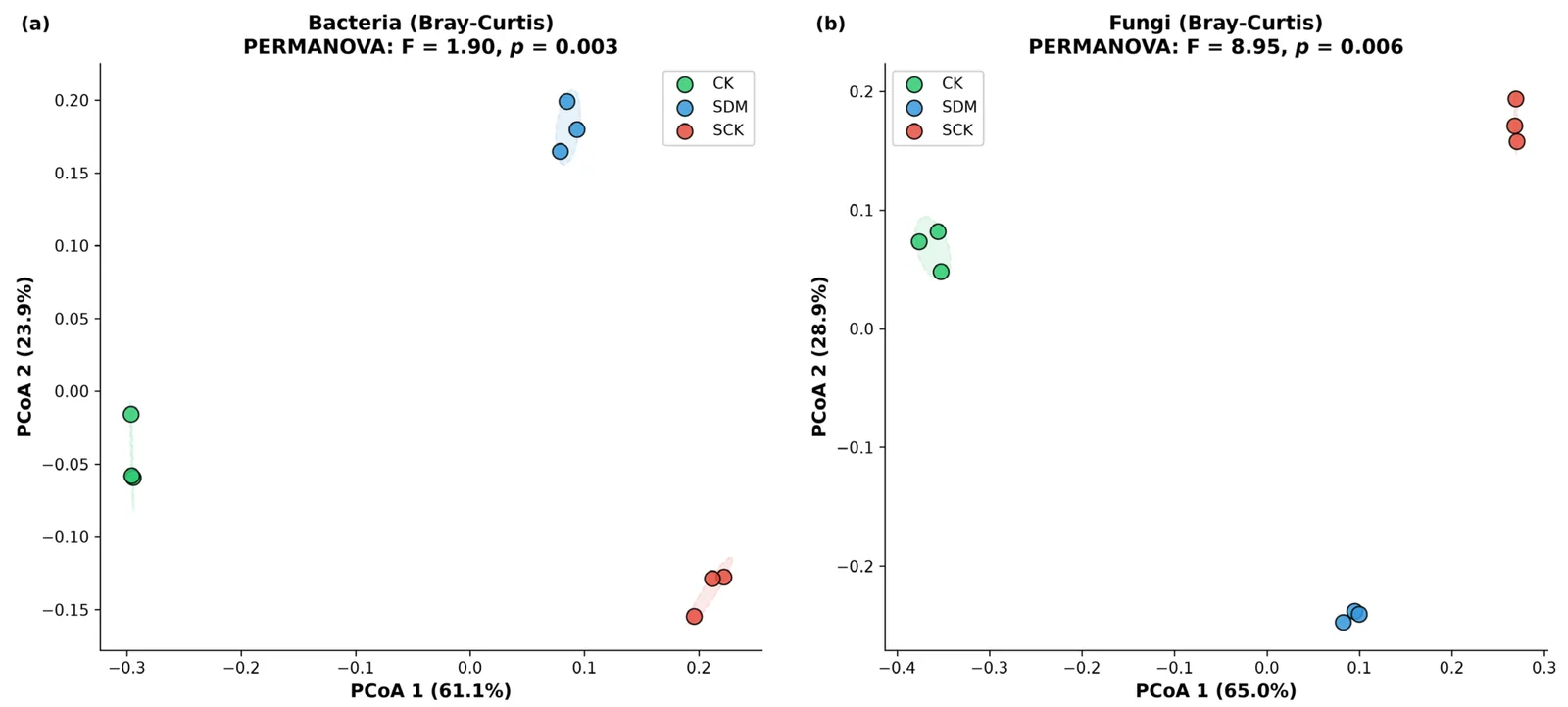
Principal coordinate analysis (PCoA) of bacterial and fungal rhizosphere communities based on Bray–Curtis distance. (**a**) Bacterial community; (**b**) fungal community. Each point represents one biological replicate (*n* = 3 per treatment). Ellipses represent 95% confidence intervals around the group centroid. PERMANOVA results (999 permutations) are shown in each panel.

Principal coordinate analysis (PCoA) based on Bray–Curtis distance revealed clear separations of microbial communities among the three treatments (Figure 3). For bacteria, the first two axes explained >50% of the total variation: CK and SCK formed distinct clusters at opposite ends of PCoA1, with SDM positioned intermediately. PERMANOVA confirmed significant differences in the bacterial community structure among treatments (F = 1.90, *p* = 0.003, 999 permutations) and the fungal community structure (F = 8.95, *p* = 0.006; 999 permutations). Fungal PCoA showed a comparable pattern, with SDM communities partially diverging from both CK and SCK.

#### 3.2.3. Community Composition at the Phylum and Genus Levels

**Figure 4:**
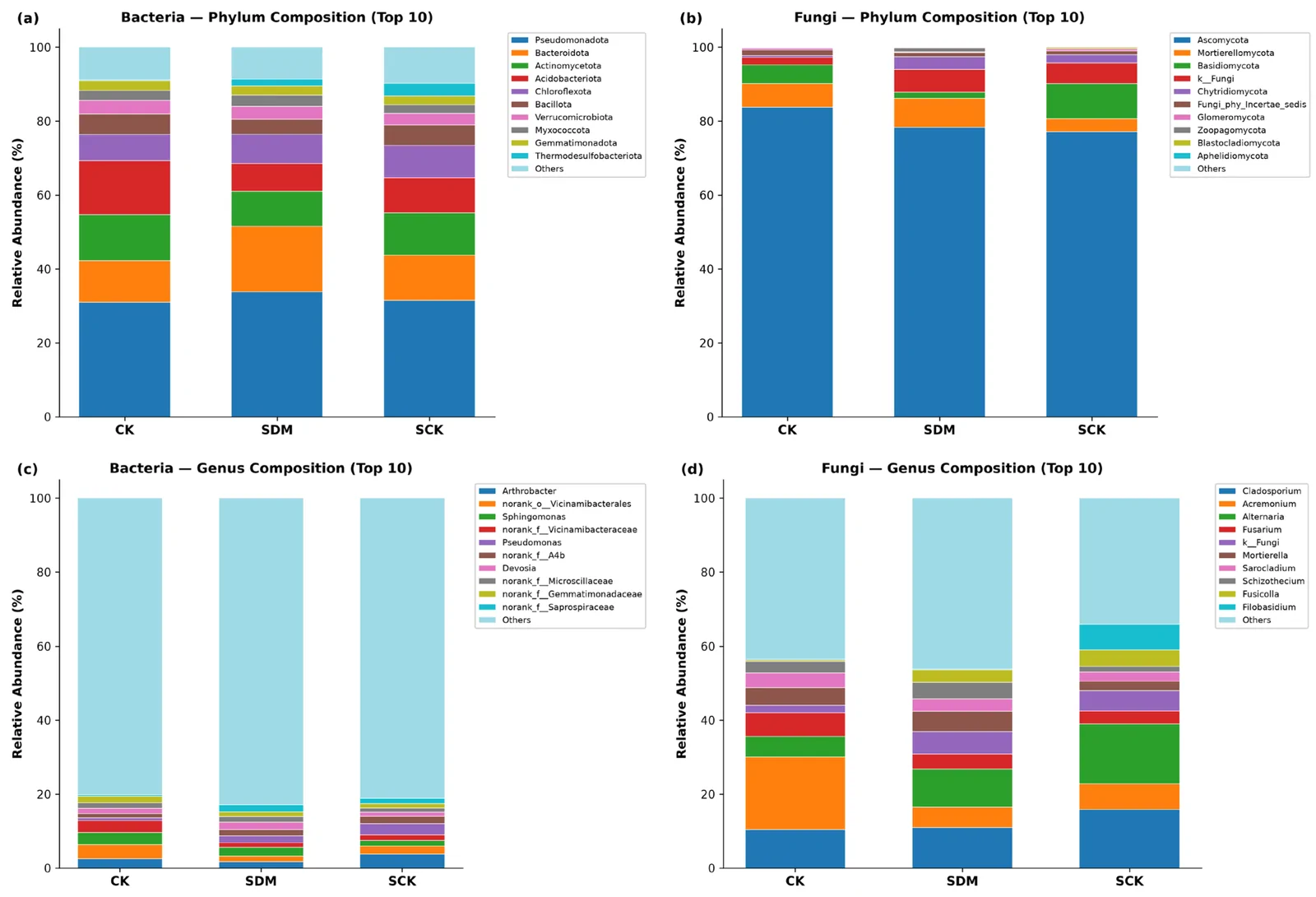
Bacterial and fungal community composition at the phylum (upper panels, top 8 phyla) and genus (lower panels, top 10 genera) levels. (**a**) Bacterial phylum-level composition; (**b**) fungal phylum-level composition; (**c**) bacterial genus-level composition; (**d**) fungal genus-level composition. Stacked bar charts show group-mean relative abundances (%) for CK, SDM, and SCK treatments (*n* = 3 per treatment). Taxa outside the top ranks are aggregated as “Others”.

Bacterial communities were dominated by eight phyla collectively accounting for >85% of relative abundance across all treatments: Pseudomonadota (31.0–33.8%), Bacteroidota (11.2–17.7%), Actinomycetota (9.5–12.5%), Acidobacteriota (7.5–14.5%), Chloroflexota (7.1–8.7%), Bacillota (4.0–5.6%), Verrucomicrobiota (3.1–3.7%), and Myxococcota (2.4–3.0%) (Figure 4, upper panels). SDM induced significant phylum-level shifts. Bacteroidota was enriched in SDM (17.7%) relative to both CK (11.2%, ** *p* < 0.01) and SCK (12.3%, ** *p* < 0.01), whereas Acidobacteriota and Actinomycetota declined under SDM compared with CK (* *p* < 0.05). Bacillota was lower in SDM (4.0%) than in CK (5.5%, ** *p* < 0.01) and SCK (5.6%, * *p* < 0.05). Pseudomonadota, the most abundant phylum, did not differ significantly among treatments, indicating that community restructuring under SDM was driven primarily by turnover among subdominant phyla.

At the genus level, SDM induced substantial compositional reorganization (Figure 4, lower panels). The ten most abundant genera with significant inter-treatment differences collectively represented approximately 14–18% of the total bacterial community. *Arthrobacter* (Actinomycetota), the most abundant genus overall, reached its highest relative abundance in SCK (3.8%), was intermediate in CK (2.5%), and was lowest in SDM (1.7%, ** *p* < 0.01 vs. SCK), indicating suppression of this halotolerant actinobacterium by the amendment. *Sphingomonas* (Alphaproteobacteria) was most abundant in CK (3.3%), intermediate in SDM (2.4%), and lowest in SCK (1.5%, ** *p* < 0.01 vs. SDM; * *p* < 0.05 vs. CK), suggesting that saline–alkali stress suppressed this genus more strongly than SDM treatment. *Pseudomonas* (Gammaproteobacteria) displayed a stress-responsive pattern: it was highest in SCK (3.0%), substantially lower in SDM (1.9%, ns vs. SCK), and lowest in CK (0.8%, *** *p* < 0.001 vs. SDM), consistent with the known proliferation of *pseudomonads* under saline conditions.

Several Vicinamibacteraceae-affiliated groups (Acidobacteriota) showed marked treatment sensitivity: the unclassified norank_f__Vicinamibacteraceae was highest in CK (3.2%) and significantly reduced in both SDM (1.3%, * *p* < 0.05) and SCK (1.6%), while norank_f__Pyrinomonadaceae followed a similar CK-dominated pattern (CK: 1.6% vs. SDM: 0.4%, * *p* < 0.05). These Acidobacteriota groups, recognized as oligotrophs adapted to low-nutrient environments, appeared sensitive to both saline stress and organic amendment. By contrast, *Devosia* (Alphaproteobacteria), a genus linked to nitrogen fixation and organic matter decomposition, was enriched in SDM (2.0%) relative to CK (1.4%, * *p* < 0.05) and SCK (1.1%, ** *p* < 0.01). *Flavobacterium* (Bacteroidota) showed the strongest SDM-associated enrichment among culturable genera, reaching 1.9% in SDM versus 0.5% in CK (** *p* < 0.01) and 0.9% in SCK (** *p* < 0.01), consistent with its role as a copiotroph responsive to organic carbon inputs. The family Saprospiraceae (Bacteroidota) was similarly enriched in SDM (1.9%) compared with CK (0.5%, ** *p* < 0.01) and SCK (1.5%, * *p* < 0.05), reinforcing the pattern of Bacteroidota-affiliated copiotroph expansion under organic amendment. *Planococcus* (Bacillota) was elevated in both SDM (1.4%) and SCK (1.7%) relative to CK (0.4%, ** *p* < 0.01 for SDM vs. CK), suggesting that this halotolerant genus responded to soil disturbance regardless of amendment type. *Metabacillus* (Bacillota) showed the most pronounced suppression under SDM (0.41%), declining by 82% relative to CK (2.28%, *** *p* < 0.001), while *Rhizobium* (Alphaproteobacteria) was also reduced in both SDM (0.8%) and SCK (0.6%) compared with CK (1.4%, * *p* < 0.05 for both). Overall, the genus-level bacterial reorganization under SDM reflected a shift from oligotrophic, CK-dominated Acidobacteriota and Bacillota lineages toward copiotrophic Bacteroidota and select Alphaproteobacteria groups with documented roles in organic matter processing.

Fungal community reorganization was equally pronounced. *Cladosporium* (Ascomycota), the most abundant fungal genus, was significantly elevated in SCK (15.9%) relative to SDM (11.0%, *** *p* < 0.001), indicating proliferation under saline–alkali stress. *Acremonium* (Ascomycota) showed an extreme CK-dominated distribution (CK: 19.7% vs. SDM: 5.4%, ** *p* < 0.01), while *Alternaria* (Ascomycota) increased under stress in SCK (16.14%), with SDM (10.4%) occupying an intermediate position (SDM vs. CK: 5.5%, * *p* < 0.05; SDM vs. SCK: * *p* < 0.05). *Fusarium* (Ascomycota), a genus encompassing both plant pathogens and saprotrophs, was most abundant in CK (6.4%) and significantly reduced in SDM (4.1%, * *p* < 0.05). Several genera were specifically enriched under SDM. *Mortierella* (Mortierellomycota), a phosphate-solubilizing fungus, reached 5.5% in SDM, significantly exceeding CK (4.8%, * *p* < 0.05) and SCK (2.5%, *** *p* < 0.001), consistent with enhanced phosphorus cycling. *Gibellulopsis* (Ascomycota) was strongly enriched in SDM (4.2%)—a 6.6-fold increase over SCK (0.6%, ** *p* < 0.01). *Schizothecium* (Ascomycota) showed a similar SDM-favoring pattern (SDM: 4.5% vs. SCK: 1.5%, ** *p* < 0.01). *Fusicolla* (Ascomycota) was virtually absent from CK soils (0.4%) but abundant in both SDM (3.4%, ** *p* < 0.01 vs. CK) and SCK (4.5%). In contrast, *Filobasidium* (Basidiomycota), a saprotrophic yeast, was dramatically enriched in SCK (7.0%) and nearly undetectable in SDM (0.2%, ** *p* < 0.01) and CK (0.3%), making it one of the strongest fungal biomarkers of the saline–alkali condition. *Plectosphaerella* (Ascomycota) was moderately abundant in SDM (2.1%) and CK (2.2%) but strongly suppressed in SCK (0.3%, ** *p* < 0.01), further underscoring the fungal community divergence between amended and unamended saline–alkali soils.

**Figure 5:**
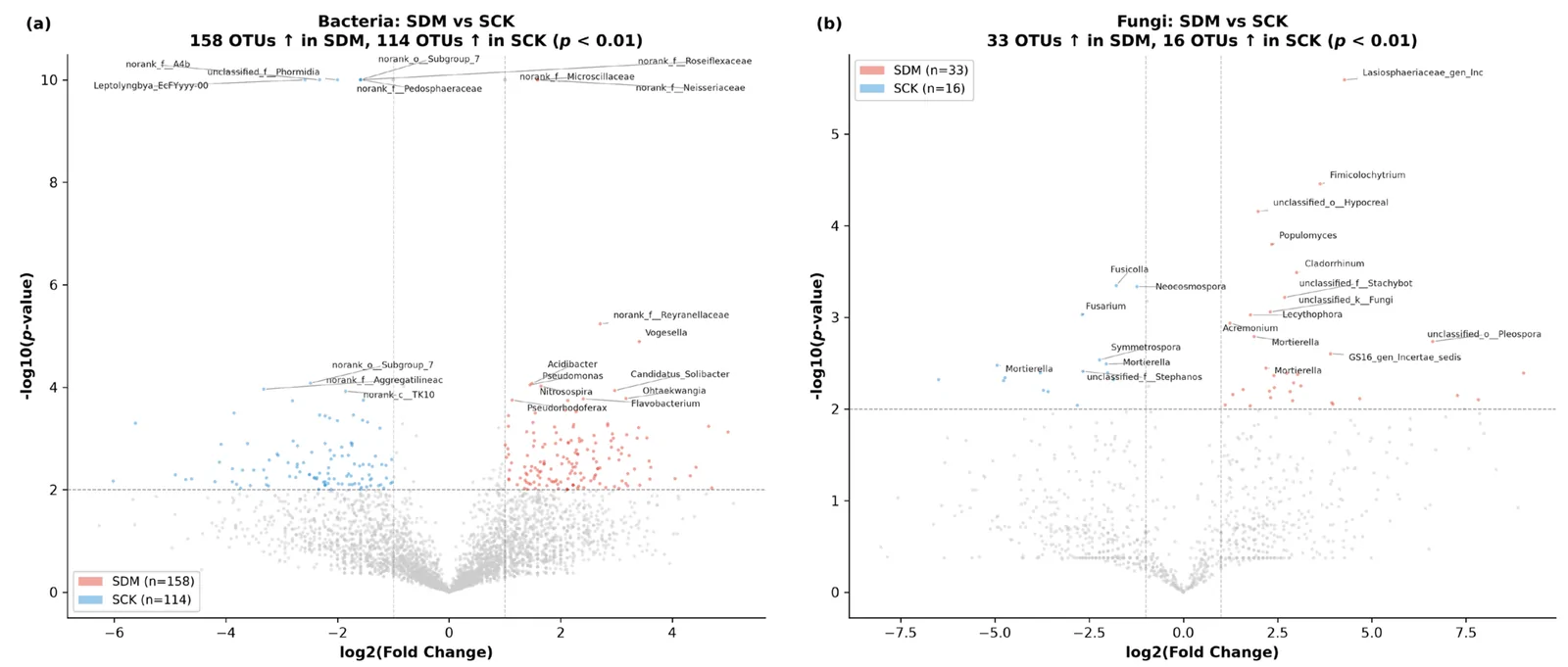
Volcano plots showing differentially abundant OTUs between SDM and SCK treatments (**a**) Bacterial community; (**b**) fungal community. Each point represents one OTU. The *x*-axis indicates log2 fold change (SDM/SCK); the *y*-axis indicates −log10(*p*-value) from Welch’s *t*-test. Horizontal dashed line: *p* = 0.01; vertical dashed lines: |log^2^FC| = 1. Red points denote OTUs significantly enriched in SDM; blue points denote OTUs significantly enriched in SCK. The top 20 most significant OTUs are labeled at the genus level. The upward arrows (↑) in the panel titles indicate the treatment group in which the listed OTUs are significantly enriched.

Differential OTU analysis between SDM and SCK identified 158 bacterial OTUs significantly enriched in SDM and 114 enriched in SCK (|log^2^FC| > 1, ** *p* < 0.01; Figure 5). The SDM-enriched fraction was dominated by OTUs affiliated with Bacteroidota (Saprospiraceae, Flavobacterium) and Alphaproteobacteria (Devosia)—taxa frequently associated with organic matter decomposition and nitrogen cycling in agricultural soils. The SCK-enriched fraction was characterized by *Pseudomonas*, *Arthrobacter*, and *Polaromonas*—genera commonly reported as halotolerant or oligotrophic taxa that proliferated under saline stress. Among fungi, 33 OTUs were enriched in SDM and 16 in SCK. Key SDM-enriched fungal genera included *Gibellulopsis* (SDM: 4.2% vs. SCK: 0.7%, ** *p* < 0.01), *Schizothecium* (SDM: 4.5% vs. SCK: 1.5%, * *p* < 0.05), and *Mortierella* (SDM: 5.5% vs. SCK: 2.5%, *** *p* < 0.001). Conversely, *Cladosporium* and *Filobasidium* were significantly more abundant in SCK (15.9% and 7.0%) than in SDM (11.0% and 0.2%, ** *p* < 0.01 for both). These patterns indicate that SDM promoted taxa involved in organic matter turnover and nutrient mobilization while suppressing the oligotrophic, stress-associated taxa characteristic of untreated saline–alkali soil.

#### 3.2.4. Environmental Drivers of Microbial Community Structure

**Figure 6:**
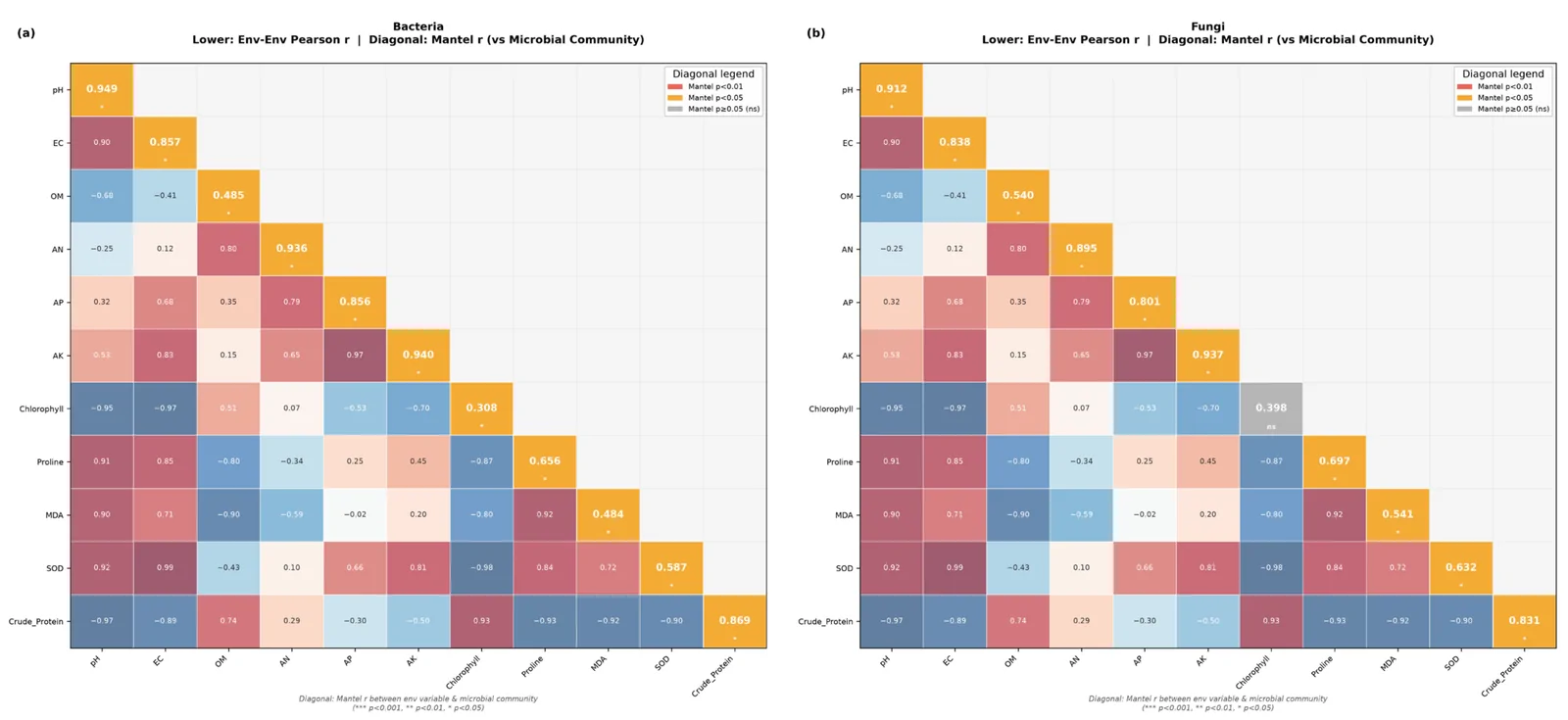
Mantel correlation heatmaps showing the association between environmental variables and rhizosphere microbial community compositions (Bray–Curtis distance, *n* = 9). (**a**) Bacterial community; (**b**) fungal community. The lower triangle displays Pearson correlation coefficients among environmental variables (RdBu color scale: blue = negative, red = positive). The diagonal cells display Mantel *r* values, color-coded by significance: red *p* < 0.01, orange *p* < 0.05, gray *p* ≥ 0.05; asterisks denote *** *p* < 0.001, ** *p* < 0.01, * *p* < 0.05. Upper triangle: blank. Environmental variables are organized into soil properties (TN–Moisture) and leaf traits (SOD–SolProt).

Mantel tests relating microbial community composition (Bray–Curtis distance) to environmental variables identified soil AK (r = 0.940), pH (r = 0.949), AN (r = 0.936), and AP (r = 0.856) as the strongest correlates of bacterial community structure (Figure 6). Leaf SOD (r = 0.587) and MDA (r = 0.484) showed moderate associations. Fungal community structure exhibited a highly congruent association pattern, with AK (r = 0.937), pH (r = 0.912), AN (r = 0.895), and AP (r = 0.801) again representing the strongest driving factors. Proline was significantly associated with fungal (r = 0.697), but not bacterial (r = 0.656, ns), community structure. The consistent primacy of soil nutrient variables—particularly AK, pH, and AP—as correlates of microbial community composition supports a mechanistic coupling between SDM-mediated soil improvements and rhizosphere microbiome remodeling.

### 3.3. Leaf Antioxidant Defense System and Biochemical Traits

**Figure 7:**
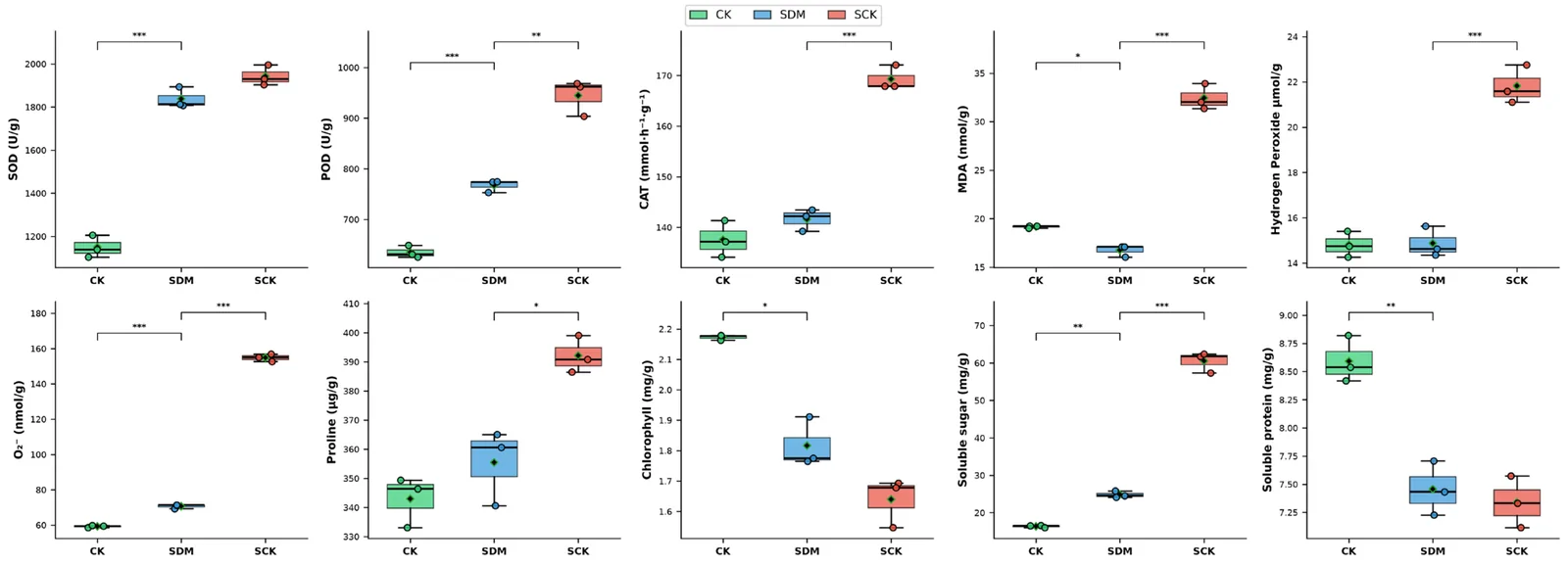
Leaf antioxidant enzyme activities, oxidative stress markers, and biochemical traits of wheat under CK, SDM, and SCK treatments. Panels display SOD, POD, CAT, MDA, H_2_O_2_, O_2_^−^, proline, chlorophyll, soluble sugars, and soluble proteins (*n* = 3 per treatment). Box elements and statistical annotations as in Figure 1.

Saline–alkali stress induced a pronounced oxidative response in wheat leaves, with SCK showing marked elevations across most stress indicators relative to CK (Table S2; Figure 7). SCK leaves exhibited the highest values for SOD (1943.11 ± 47.60 U/g vs. CK: 1149.81 ± 51.39 U/g), POD (944.89 ± 35.67 U/g vs. CK: 635.14 ± 11.90 U/g), CAT (169.30 ± 2.44 vs. CK: 137.57 ± 3.63 mmol/h/g), MDA (32.45 ± 1.33 vs. CK: 19.17 ± 0.13 nmol/g), H_2_O_2_ (21.82 ± 0.84 vs. CK: 14.81 ± 0.57 μmol/g), O_2_^−^ (154.90 ± 2.13 vs. CK: 59.29 ± 0.58 nmol/g), and soluble sugars (60.50 ± 2.76 vs. CK: 16.39 ± 0.29 mg/g). Chlorophyll was significantly depressed in SCK (1.64 ± 0.08 vs. CK: 2.17 ± 0.01 mg/g).

Under SDM, leaf antioxidant enzyme activities were substantially elevated relative to CK: SOD reached 1838.02 ± 48.19 U/g (*** *p* < 0.001 vs. CK, +59.9%; ns vs. SCK, −5.4%), and POD increased to 767.46 ± 12.21 U/g (*** *p* < 0.001 vs. CK, +20.8%; ** *p* < 0.01 vs. SCK, −18.8%). CAT activity in SDM (141.62 ± 2.18 mmol/h/g) was comparable to CK (ns, +2.9%) and significantly lower than SCK (*** *p* < 0.001, −16.3%).

SDM effectively suppressed oxidative damage markers. MDA content in SDM leaves (16.75 ± 0.60 nmol/g) was the lowest among all treatments: 12.6% below CK (* *p* < 0.05) and 48.4% below SCK (*** *p* < 0.001). H_2_O_2_ in SDM (14.87 ± 0.67 μmol/g) was fully normalized to CK levels (ns, +0.5%) and 31.8% lower than SCK (*** *p* < 0.001). Superoxide anions in SDM (70.68 ± 1.17 nmol/g) were reduced by 54.4% compared to SCK (*** *p* < 0.001) but remained 19.2% above CK (*** *p* < 0.001).

Leaf chlorophyll under SDM (1.82 ± 0.08 mg/g) did not recover to CK levels (* *p* < 0.05, −16.4%), though it trended higher than SCK (ns, +10.8%). Soluble sugars in SDM leaves (24.82 ± 0.87 mg/g) was intermediate—above CK (+51.5%, ** *p* < 0.01) but far below the elevated SCK level (−59.0%, *** *p* < 0.001). Soluble proteins in SDM (7.46 ± 0.24 mg/g) were lower than CK (** *p* < 0.01, −13.2%) and comparable to SCK (ns). Proline in SDM leaves (355.45 ± 13.01 μg/g) was moderately lower than SCK (392.15 ± 6.41 μg/g, * *p* < 0.05, −9.4%) and comparable to CK (342.97 ± 8.66 μg/g, ns).

### 3.4. Grain Physiological and Biochemical Parameters

**Figure 8:**
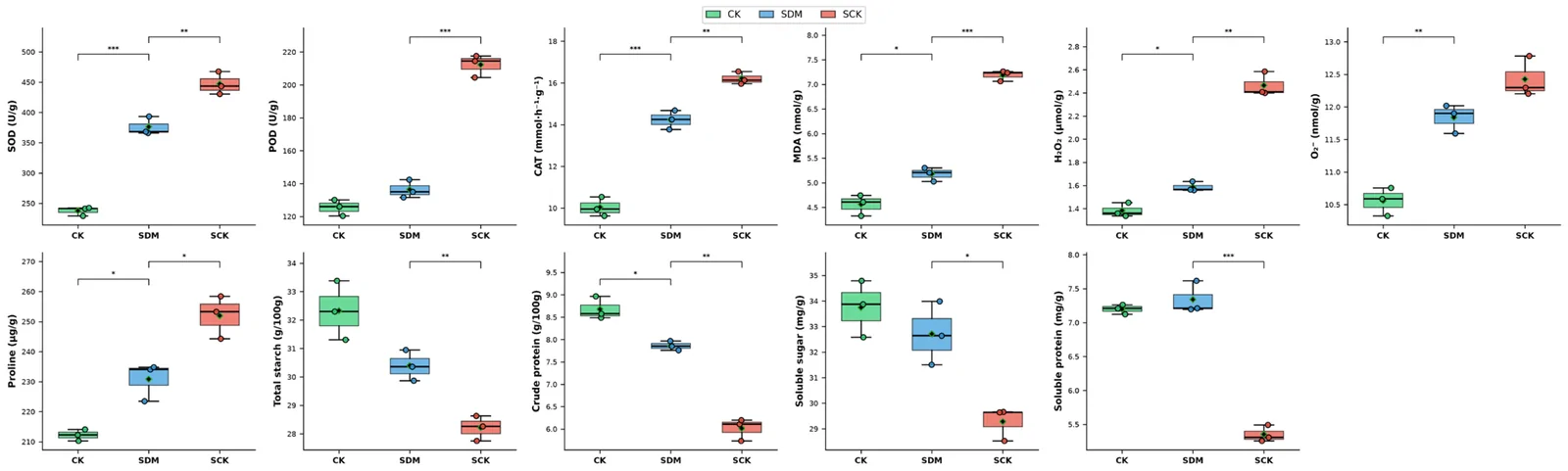
Grain antioxidant enzyme activities, oxidative stress markers, and quality parameters of wheat under CK, SDM, and SCK treatments. Panels display SOD, POD, CAT, MDA, H_2_O_2_, O_2_^−^, proline, total starch, crude protein, soluble sugars, and soluble proteins (*n* = 3 per treatment). Box elements and statistical annotations as in Figure 1.

Grain biochemical profiles differed markedly from leaf responses (Table S3; Figure 8). CK grains had the lowest MDA content (4.56 ± 0.21 nmol/g) among all treatments. SDM grain MDA (5.18 ± 0.14 nmol/g) was moderately elevated relative to CK (* *p* < 0.05, +13.6%), yet substantially lower than SCK (7.19 ± 0.11 nmol/g, *** *p* < 0.001, −28.0%).

Grain antioxidant enzyme activities in SDM were consistently intermediate between CK and SCK. SOD in SDM grains (376.01 ± 15.11 U/g) exceeded CK (237.76 ± 7.40 U/g, *** *p* < 0.001, +58.1%) but fell below SCK (447.19 ± 18.91 U/g, ** *p* < 0.01, −15.9%). POD followed a similar intermediate pattern (SDM: 136.42 ± 5.53 U/g; CK: 125.56 ± 4.93 U/g, ns; SCK: 212.24 ± 6.82 U/g, *** *p* < 0.001, −35.7%). CAT in SDM grains (14.24 ± 0.45 mmol/h/g) was elevated relative to CK (10.04 ± 0.46 mmol/h/g, *** *p* < 0.001, +41.8%) and reduced relative to SCK (16.22 ± 0.30 mmol/h/g, ** *p* < 0.01, −12.2%).

H_2_O_2_ in SDM grains (1.59 ± 0.04 μmol/g) was moderately above CK (1.38 ± 0.06 μmol/g, * *p* < 0.05, +14.8%) but 35.6% below SCK (2.47 ± 0.10 μmol/g, ** *p* < 0.01). The superoxide anions in SDM grains (11.84 ± 0.22 nmol/g) were significantly elevated versus CK (10.56 ± 0.22 nmol/g, ** *p* < 0.01, +12.1%) and comparable to SCK (12.43 ± 0.31 nmol/g, ns).

Grain quality parameters showed partial normalization under SDM. Crude protein in SDM grains (7.86 ± 0.10 g/100 g) was slightly below CK (8.68 ± 0.26 g/100 g, * *p* < 0.05, −9.4%) but 30.7% above SCK (6.02 ± 0.25 g/100 g, ** *p* < 0.01). Soluble sugars in SDM grains (32.71 ± 1.25 mg/g) were comparable to CK (33.75 ± 1.11 mg/g, ns) and moderately elevated relative to SCK (29.28 ± 0.65 mg/g, * *p* < 0.05, +11.7%). Total starch in SDM grains (30.39 ± 0.54 g/100 g) was slightly below CK (32.33 ± 1.04 g/100 g, ns) but exceeded SCK (28.21 ± 0.44 g/100 g, ** *p* < 0.01, +7.7%). Soluble proteins in SDM grains (7.34 ± 0.24 mg/g) were comparable to CK (7.20 ± 0.07 mg/g, ns) and significantly higher than SCK (5.35 ± 0.12 mg/g, *** *p* < 0.001, +37.1%). Proline in SDM grains (230.83 ± 6.33 μg/g) was moderately elevated versus CK (212.26 ± 1.91 μg/g, * *p* < 0.05) and slightly below SCK (252.00 ± 7.15 μg/g, * *p* < 0.05).

## 4. Discussion

### 4.1. Soil Nutrient Enhancement and Microbiome Remodeling Drive Stress Alleviation

The combination of organic fertilizer and microbial inoculant (SDM) produced substantial improvements in soil fertility—most notably the near-doubling of available phosphorus (+96.0% vs. SCK) and the 51.8% increase in available potassium—that were closely associated with rhizosphere microbiome restructuring. Mantel tests identified soil AK, pH, and AN as the dominant environmental correlates of both bacterial and fungal community compositions, suggesting that nutrient enrichment and microbiome reassembly were closely associated, although the correlational design does not establish directionality between soil chemistry and community shifts. A recent global meta-analysis found that organic amendments consistently outperformed chemical fertilizers in stimulating soil phosphatase activity and phoD-harboring bacterial communities, offering a mechanistic explanation for the large AP increase we observed [26]. The concurrent rise in bacterial and fungal Shannon diversity under SDM is consistent with evidence that organic inputs relax the strong environmental filtering imposed by salinity, permitting more diverse microbial assemblages to be established [27]. In saline–alkali soils, biochar and organic fertilizer co-application has been shown to increase copiotrophic phyla (Proteobacteria, Bacteroidota) at the expense of oligotrophic taxa—a shift mirrored in the present study [28]. Meta-omics analyses further demonstrate that organic–mineral co-application enriches functional genes involved in nitrogen fixation (nifH), nitrification (amoA), and alkaline phosphatase (phoD) in saline–alkali rhizospheres, directly linking compositional changes to enhanced nutrient-cycling capacity.

PCoA placed SDM communities intermediately between CK and SCK, suggesting a partial transition of the rhizosphere microbiome away from the saline–alkali state. This is consistent with the emerging concept of microbiome engineering via resource-driven community assembly: labile carbon from organic amendments reduces the competitive advantage of oligotrophic, stress-tolerant taxa and allows copiotrophic functional groups to expand [29,30].

Genus-level reorganization revealed ecologically coherent patterns that linked community structure to soil function. *Flavobacterium* and the *Saprospiraceae* (Bacteroidota)—both well-characterized copiotrophs that degrade complex organic polymers including proteins, carbohydrates, and cellulose—expanded markedly under SDM, providing a microbial explanation for the enhanced organic matter mineralization we documented. Bacteroidota members are among the primary responders to labile carbon inputs in soil, and their increase from 11.2% to 17.7% of relative abundance constitutes a substantial community-level shift. Lignocellulose degradation in organic amendments is mediated by synergistic bacterial–fungal consortia that produce extracellular hydrolytic enzymes, a process accelerated by *Bacillus* inoculation during composting [31,32]. Co-inoculation with lignocellulose-degrading microorganisms during composting enhances dissolved organic matter transformation and promotes stable humic substance formation, which serves as a long-term reservoir of soil organic carbon and nitrogen [33]. The carbon-to-nitrogen ratio and ammonium-to-nitrate balance are critical regulators of organic nitrogen fraction dynamics and humification efficiency during agricultural waste composting, directly affecting amendment quality and stability [34]. The drivers of lignocellulose-degrading enzyme gene abundance and humic acid formation during composting are shaped by microbial community composition and Fenton-like pretreatment conditions [35,36]. *Devosia* (Alphaproteobacteria), significantly enriched in SDM (2.0% vs. 1.1% in SCK), includes species capable of nitrogen fixation and denitrification, potentially contributing to the enhanced soil nitrogen pools (TN +23.6%, AN +33.5% vs. SCK) through both symbiotic and free-living pathways [37]. The enrichment of Sphingomonas under SDM (2.4% vs. 1.5% in SCK) is noteworthy, as members of this genus produce plant growth-promoting phytohormones including gibberellins and indole-3-acetic acid, suggesting a possible hormonal link between rhizosphere microbiome composition and plant performance [38].

SDM also suppressed several genera characteristic of the saline–alkali environment. *Arthrobacter*, the most abundant bacterial genus overall, was reduced by 56% under SDM (1.7% vs. 3.8% in SCK). Although *Arthrobacter* species are metabolically versatile and stress-tolerant—with documented resistance to desiccation and high osmolarity—their dominance in SCK soils likely reflects the competitive advantage of oligotrophic survival strategies under resource-limited saline conditions [39]. *Pseudomonas*, another genus enriched in SCK (3.0% vs. 1.9% in SDM), includes species that thrive at high salinity through compatible solute accumulation and Na^+^ efflux mechanisms; its partial normalization under SDM represents a microbial signature of amelioration. The largest relative suppression was in Metabacillus, which fell from 2.3% in CK to 0.4% in SDM—an 82% decline. Given that *Metabacillus* species are generally considered as plant-beneficial rhizobacteria capable of nitrogen fixation and phytohormone production, this unexpected decline may reflect competitive exclusion by rapidly proliferating copiotrophs that are better adapted to the organic-matter-enriched SDM soil or reduced plant recruitment of these mutualists under improved nutrient conditions.

Fungal genus-level shifts provided complementary evidence for enhanced nutrient cycling. *Mortierella*—a well-documented phosphate-solubilizing fungus that secretes organic acids (citrate, oxalate, malate) to mobilize insoluble soil phosphates—was enriched in SDM (5.5% vs. 2.5% in SCK, *p* < 0.001), offering a microbial mechanism consistent with the 96.0% increase in soil AP [40,41]. The concurrent enrichment of *Gibellulopsis* (4.2% vs. 0.7% in SCK) and *Schizothecium* (4.5% vs. 1.6%), both saprotrophic Ascomycota capable of lignocellulose degradation, indicates that SDM fostered a fungal community oriented toward organic matter decomposition—consistent with the increases in soil OM (+24.1%) and nutrient availability. Conversely, SDM suppressed several fungal taxa characteristics of the saline–alkali environment: *Filobasidium*, a basidiomycetous yeast, was 29-fold more abundant in SCK (7.0%) than in SDM (0.2%), making it a robust fungal biomarker of saline–alkali stress and *Cladosporium* and *Alternaria*, both stress-tolerant Ascomycota, were reduced by 31% and 36%, respectively, under SDM [42]. The reduction in *Fusarium* abundance under SDM (4.1% vs. 6.4% in CK) may carry additional agronomic significance, since several Fusarium species are recognized cereal pathogens whose suppression could benefit root and crown health under field conditions [43].

We emphasize that the ecological functions attributed to the individual genera above are inferred from the published literature and represent plausible—not confirmed—capacities in the present soil system. Many traits relevant to nutrient cycling, plant growth promotion, and stress tolerance are strain-specific rather than genus-conserved and cannot be reliably predicted from 16S rRNA or ITS amplicon data alone. Metagenomic, metatranscriptomic, or culture-based approaches would be required to verify the functional contributions hypothesized here.

Comparable phylum-level shifts—copiotroph expansion at the expense of oligotrophic halophiles following organic matter inputs—have been documented in other saline–alkali soil remediation studies [44]. Halotolerant plant-growth-promoting rhizobacteria (HT-PGPR) are particularly relevant in this context: they not only tolerate saline conditions but actively secrete ACC deaminase, IAA, and exopolysaccharides that improve root architecture and soil aggregation while reducing plant ethylene levels [45,46,47]. The genus-level signatures identified here—the enrichment of organic matter degraders (*Flavobacterium*, *Saprospiraceae*, *Schizothecium*), phosphate solubilizers (*Mortierella*), and nitrogen-cycling taxa (*Devosia*), alongside the suppression of stress-associated genera (*Arthrobacter*, *Pseudomonas*, *Filobasidium*, *Cladosporium*)—collectively describe a pattern of microbiome-mediated soil remediation in which organic amendment redirects community assembly away from oligotrophic survival strategies and toward nutrient-cycling functional guilds. The composting of agricultural organic wastes involves dynamic microbial succession, with functional keystone taxa driving nitrogen conversion and humic substance formation; Bacillus species contribute pivotally through lignocellulolytic enzyme secretion and the promotion of humification [48,49]. During composting, mineral additives such as ferrous ions, calcium superphosphate, and zeolite further enhance nitrogen retention, phosphatase activity, and phosphorus availability in the final compost product.

Enhanced K^+^ availability is especially critical under saline–alkali stress: K^+^ may compete with Na^+^ at root uptake sites and potentially support osmotic adjustment and Na^+^ exclusion through pathways such as the SOS signaling cascade, although tissue ion concentrations were not measured in this study [50,51]. The moderate but significant reductions in pH (−2.5%) and EC (−5.8%) further alleviated rhizosphere ionic stress and likely improved micronutrient bioavailability [52]. The OM increase (+24.1% vs. SCK) promoted soil aggregation and water retention while supplying substrates for heterotrophic microbial metabolism [53]. Organic amendments—including humic acid, vermicompost, and microbial organic fertilizers—consistently reduce soil pH and EC in saline–alkali field trials, mediated by organic acid production during decomposition and enhanced Ca^2+^–Na^+^ exchange at soil colloid surfaces [54,55]. Fermentation wastewater and related organic byproducts can also serve as fertilizers that modify soil microbial community structure while delivering plant-available nutrients [56]. Biochar and clay mineral amendments combined with organic composting can immobilize heavy metals while promoting functional bacterial communities involved in organic fraction transformation—a dual benefit for soil remediation and fertility. Together, the soil and microbiome data support a model in which organic matter incorporation and microbial inoculant activity synergistically improve nutrient availability, reshape the rhizosphere microbiome, and establish a less hostile root environment. Temperature variation and the balance of nitrate to ammonium shape microbial community dynamics and greenhouse gas emissions during organic waste processing, with nitrifying and denitrifying functional guilds responding in distinct and predictable patterns [57,58,59]. Inoculation with anti-acidification microbial consortia during composting redirects carbohydrate metabolism in keystone microbes, accelerating the transition from labile to stable organic matter pools [60]. The consistent CK < SDM < SCK gradient observed across antioxidant enzyme activities, oxidative damage markers, and osmolyte levels in both leaf and grain tissues confirms that the physiological responses reported here are driven by saline–alkali stress severity rather than by treatment-specific artifacts.

### 4.2. Leaf Responses: Microbiome-Supported Antioxidant Activation and Metabolic Adjustment

Leaf antioxidant profiles under SDM reflected a state of controlled activation: SOD (+59.9%) and POD (+20.8%) were robustly upregulated relative to CK, yet remained below extreme SCK levels. This intermediate enzymatic positioning, combined with the lowest MDA content observed across all treatments (−48.4% vs. SCK, −12.6% vs. CK), suggests that SDM plants mounted an effective defense without incurring the full metabolic costs of maximal enzyme induction. The Mantel correlation between leaf SOD and microbial community structure (bacteria r = 0.587) supports a model in which the rhizosphere microbiome contributes to leaf-level oxidative homeostasis, likely through microbially mediated improvements in nutrient acquisition—particularly P and K—that support antioxidant enzyme biosynthesis [61,62]. Exogenous phytohormones and signaling molecules can enhance plant antioxidant capacity by upregulating the ascorbate–glutathione cycle and osmolyte accumulation, representing a parallel strategy to microbial-mediated oxidative protection [63,64]. Microbial inoculants with ACC deaminase activity reduce stress ethylene production under saline conditions, which in turn alleviates ROS overproduction and lessens the demand for maximal antioxidant enzyme induction [65,66]. Leaf CAT was restored to CK levels (ns), a finding that can be understood through the functional redundancy between CAT and the ascorbate–glutathione (AsA-GSH) pathway in H_2_O_2_ processing: under moderate H_2_O_2_ loads, the AsA-GSH cycle and class III peroxidases predominate, with CAT functioning as a peroxisomal safety valve activated only under high H_2_O_2_ fluxes [67]. The residual elevation of O_2_^−^ (+19.2% vs. CK) may reflect steady-state generation by the enlarged SOD pool rather than pathological oxidative accumulation.

Carbon metabolism displayed classical stress signatures that SDM partially reversed. SCK leaves accumulated extreme levels of soluble sugars (60.50 mg/g, 3.7× CK), driven by impaired phloem export under osmotic stress—consistent with the documented disruption of sucrose transporter expression and phloem loading under saline conditions [68]. SDM reduced leaf soluble sugars to 24.82 mg/g (−59.0% vs. SCK), indicating substantially improved photosynthate export from source leaves. The lack of chlorophyll recovery under SDM (ns vs. SCK) suggests that salinity-driven pigment degradation proceeds through mechanisms—likely Na^+^-induced Mg^2+^ displacement and ROS-mediated thylakoid damage—that are not readily reversed by nutrient improvement alone [69]. The limited proline accumulation across treatments (CK: 343 → SCK: 392 → SDM: 355 μg/g), in the context of effective oxidative protection, aligns with the emerging view that certain wheat genotypes rely on proline-independent salt tolerance pathways involving glycine betaine, sugar alcohols, and integrated ion transport mechanisms [70]. This strategy may be reinforced by improved K^+^/Na^+^ homeostasis and rhizosphere microbial buffering under SDM. Compatible solute application has been shown to enhance salt tolerance in wheat primarily through non-proline routes—including trehalose and glycine betaine accumulation—which are energetically more favorable under sustained moderate stress [71].

### 4.3. Grain Quality: Partial Restoration Through Upstream Stress Buffering

Grain biochemical profiles were fundamentally distinct from leaf responses, with antioxidant enzymes consistently positioned between CK and SCK levels. This pattern likely reflects effective upstream buffering: the potential for root-level K^+^-mediated Na^+^ exclusion, leaf-level ROS detoxification, and microbiome-mediated rhizosphere amelioration collectively attenuated the stress signal reaching developing grains [72]. The contrasting CAT profiles—normalized in leaves yet elevated 41.8% above CK in grains—illustrate tissue-specific H_2_O_2_-scavenging strategies. Kinetic modeling has demonstrated that the balance between CAT and the AsA-GSH pathway in H_2_O_2_ processing is highly context-dependent: in tissues with low peroxisomal densities, such as developing cereal grains, CAT assumes a proportionally greater role in H_2_O_2_ homeostasis [73].

CK grains consistently exhibited the lowest MDA (4.56 nmol/g) and the highest quality parameters—8.68 g/100 g of crude protein, 33.75 mg/g of soluble sugars, and 32.33 g/100 g of total starch —confirming that non-saline conditions maximize storage compound deposition, consistent with field studies showing that optimized soil conditions and tillage management enhance wheat grain yield, biomass accumulation, and nitrogen remobilization [74]. The efficient enzymatic conversion of wheat straw—through alkaline peroxide pretreatment and cellulase hydrolysis—illustrates the substantial carbohydrate reservoir in wheat biomass that, under optimal growth conditions, is preferentially partitioned to grain starch rather than structural lignocellulose [75]. SDM shifted grain composition substantially toward CK values (soluble sugars: 32.71 mg/g, ns vs. CK; starch: 30.39 g/100 g, ns; soluble proteins: 7.34 mg/g, ns vs. CK), though a residual gap in crude protein persisted (−9.4% vs. CK). The concurrence of reduced leaf soluble sugars (−59.0% vs. SCK) with normalized grain soluble sugars is consistent with improved phloem loading and source-to-sink carbon transport contributing to grain quality improvements [76]. Recent work on source–sink dynamics in cereals has established that the capacity for sucrose export from flag leaves—mediated by SUT and SWEET transporter families—is a primary determinant of the grain-filling rate and final starch and protein content under abiotic stress [77]. Partial rather than complete protein restoration may reflect the persistent metabolic demand for N-containing compatible solutes and stress-responsive proteins in vegetative tissues, which competes with N allocation to grain storage proteins [78]. Organic amendment strategies that combine microbial inoculants with optimized N supply achieve superior grain protein content relative to organic amendments alone, suggesting that the moderate residual protein deficit under SDM could be addressed by fine-tuning the N component of the amendment formulation [79].

A crude protein content of 9.4% below that of the non-saline control is noteworthy from an end-use perspective, as grain protein concentration is a key determinant of wheat quality grade and market value. The approximately 30% increase over SCK (6.02 g/100 g) represents a meaningful improvement under saline conditions, but whether the residual deficit relative to CK is agronomically consequential depends on the end-use class of the wheat and local quality grading thresholds. The suggestion to fine-tune the N component of the amendment formulation is part of ongoing field trials designed to test whether moderate nitrogen supplementation can close the remaining protein gap without compromising the oxidative protection and starch accumulation benefits achieved under SDM.

The moderate MDA elevation (+13.6% vs. CK) and sub-threshold ROS levels in SDM grains should be interpreted with care. Rather than indicating pathological stress, these moderate ROS concentrations may participate in the physiological regulation of grain development—including the modulation of endosperm cell division, starch synthase activity, and programmed cell death during grain maturation—as documented in developmental studies of cereal caryopsis [80]. The recognition that ROS function as dual-role molecules (damaging at high concentrations, but developmental signals at moderate concentrations) provides a framework for understanding why SDM grains achieve near-normal quality parameters despite a detectable oxidative signature. Proline, as in leaves, followed the rank order SCK > SDM > CK with limited magnitude, further supporting the interpretation that this cultivar largely employed proline-independent osmoregulation. Genome-wide analyses of auxin-responsive gene families in wheat have identified key regulatory loci controlling developmental transitions and stress responses, underscoring the genetic complexity underlying the multi-tissue phenotypes observed here [81].

### 4.4. Limitations and Future Directions

Several limitations warrant mention. The replication level (*n* = 3) limits statistical power for detecting moderate effect sizes, and sampling at a single time point (maturity) precludes the temporal analysis of microbial succession and antioxidant dynamics across developmental stages. Although Mantel analyses revealed strong correlations between soil variables and microbial community structure, correlation alone does not establish causation—targeted manipulative experiments using synthetic microbial communities or functional gene knockouts are needed to validate the proposed microbiome-mediated mechanisms [82]. The specific microbial taxa and functional genes (e.g., phoD, gcd, nifH) responsible for enhanced P, K, and N mobilization were not resolved through metagenomic sequencing, which would strengthen mechanistic inference linking community shifts to soil nutrient improvements. Baseline soil chemical data prior to treatment application were not collected; however, the randomized complete block design on adjacent fields with shared parent material and cultivation history, together with the low post-harvest variability in treatment-insensitive parameters such as total K (CV < 5% within saline–alkali plots), provides indirect support for pretreatment homogeneity within each salinity class.

Furthermore, although CK and SCK plots were established on adjacent fields sharing the same fluvo-aquic soil parent material, pre-existing differences in soil properties beyond salinity (e.g., texture, organic matter history, prior management) cannot be fully excluded; this represents an additional limitation for attributing CK-vs-SCK differences solely to saline–alkali. Tissue ion concentrations (Na^+^, K^+^, Ca^2+^) were not measured, precluding direct assessment of the ionic homeostasis hypothesis. Finally, multi-location field validation is essential for translating these findings into practical recommendations for saline–alkali wheat production [83].

The SDM treatment combined decomposed sheep manure with a defined microbial inoculant, and the present experimental design did not allow the effects of the introduced microbial strains to be distinguished from those of the manure matrix and its endogenous microbial community. Well-composted manure harbors diverse microorganisms that may themselves contribute to the observed shifts in soil nutrient availability and rhizosphere microbiome structure. A manure-only control treatment would be necessary to disentangle these effects. This limitation is acknowledged and will be addressed in future work through the inclusion of appropriate factorial controls.

## 5. Conclusions

This study presents a comprehensive, multi-tissue analysis of how an organic fertilizer–microbial inoculant combination (SDM) affects wheat grown under saline–alkali stress. SDM substantially improved soil fertility, with available phosphorus increasing by 96.0% and available potassium by 51.8% relative to the saline–alkali control. These soil improvements coincided with rhizosphere microbiome remodeling characterized by the enrichment of copiotrophic, nutrient-cycling taxa—including Flavobacterium, Devosia, Mortierella, and Gibellulopsis—and the suppression of halotolerant, stress-associated genera such as *Arthrobacter*, *Pseudomonas*, and *Filobasidium*. Mantel tests identified soil available potassium, pH, and available nitrogen as the strongest environmental correlates of microbial community structure, establishing a strong quantitative association between SDM-mediated soil amelioration and microbiome reassembly.

These microbiome shifts translated into coordinated physiological benefits at the plant level. Leaf antioxidant enzymes were activated to an intermediate level—above the non-saline control but below the extreme saline–alkali condition—producing the lowest malondialdehyde content across all treatments. This controlled activation profile suggests that SDM enabled effective oxidative protection without incurring the full metabolic cost of maximal enzyme induction. Grain quality parameters, including soluble sugars and total starch, were restored to levels statistically indistinguishable from non-saline control grains, while crude protein showed partial but substantial recovery. The concurrent normalization of leaf soluble sugars and grain storage compounds is consistent with improved source-to-sink carbon transport under SDM treatment.

In summary, our findings establish a coordinated soil–microbiome–plant response pattern in which organic–microbial amendment simultaneously enhances soil nutrient availability, redirects rhizosphere community assembly from stress-tolerant oligotrophs toward nutrient-cycling functional guilds, and produces coordinated improvements in leaf oxidative homeostasis and grain nutritional quality. These results offer a conceptual framework for developing microbiome-informed management strategies for saline–alkali wheat production. Future studies incorporating factorial controls (manure-only and inoculant-only treatments), metagenomic and metatranscriptomic approaches, multi-location field validation, and temporal sampling across the full wheat growth cycle will be essential to resolve the contributions of specific treatment components and the microbial functional mechanisms identified here and to translate these findings into practical agronomic recommendations.

## Data Availability

The raw sequencing data generated in this study have been deposited in the NCBI Sequence Read Archive (SRA) and are publicly available under BioProject accession number PRJNA1484213. The dataset is fully accessible without any restriction.

## Supplementary Materials

The following supporting information can be downloaded at https://www.mdpi.com/article/10.3390/biology15171467/s1, Table S1. Soil physicochemical properties; Table S2. Leaf antioxidant enzyme activities, oxidative stress markers, and biochemical traits of wheat; Table S3. Grain antioxidant profiles, oxidative stress markers, and quality parameters of wheat; Table S4. Sequencing and rarefaction statistics for 16S rRNA and ITS amplicon libraries.
