# Supplementary material for: Organic Fertilizer Combined with Microbial Inoculant Alleviates Saline–Alkali Stress in Wheat: A Multi-Tissue Analysis
Source: Biology (Basel). 2026 Aug 29;15(17):1467. doi: 10.3390/biology15171467 (PMC13565493; doi:10.3390/biology15171467)
Supplement: Supplementary file 1 [file biology-15-01467-s001.zip › biology-4492586-supplementary.pdf]

Table S1. Soil physicochemical properties

Indicator	CK	SDM	SCK
TN (g/kg)	0.82 ± 0.01 b	0.92 ± 0.00 c	0.75 ± 0.01 a
TP (g/kg)	1.06 ± 0.00 a	1.04 ± 0.00 a	1.04 ± 0.01 a
TK (g/kg)	9.74 ± 0.16 b	9.73 ± 0.06 b	9.46 ± 0.11 a
AN (mg/kg)	75.52 ± 2.30 a	96.80 ± 2.11 b	72.48 ± 1.06 a
AP (mg/kg)	6.00 ± 0.14 a	20.72 ± 0.37 c	10.57 ± 0.42 b
AK (mg/kg)	102.28 ± 0.96 a	282.89 ± 1.42 c	186.30 ± 1.46 b
OM (g/kg)	10.64 ± 0.27 b	11.75 ± 0.46 c	9.47 ± 0.18 a
pH	8.15 ± 0.04 a	8.26 ± 0.03 b	8.47 ± 0.04 c
EC (µS/cm)	153.37 ± 0.97 a	370.33 ± 5.51 b	393.33 ± 4.04 c
Moisture	0.96 ± 0.01 a	0.90 ± 0.05 a	1.08 ± 0.01 b

Values are mean ± SD (n = 3). CK (non-saline control), SDM (organic fertilizer + microbial inoculant), and SCK (saline-alkali control). Different lowercase letters within a row denote significant differences among treatments (Welch's t-test, $p < 0.05$). TN, total nitrogen; TP, total phosphorus; TK, total potassium; AN, alkaline hydrolyzable nitrogen; AP, available phosphorus; AK, available potassium; OM, organic matter; EC, electrical conductivity.

Table S2. Leaf antioxidant enzyme activities, oxidative stress markers, and biochemical traits of wheat

**Indicator**	**CK**	**SDM**	**SCK**
SOD (U/g)	1149.81 ± 51.39 a	1838.02 ± 48.19 b	1943.11 ± 47.60 b
POD (U/g)	635.14 ± 11.90 a	767.46 ± 12.21 b	944.89 ± 35.67 c
CAT (mmol/h/g)	137.57 ± 3.63 a	141.62 ± 2.18 a	169.30 ± 2.44 b
MDA (nmol/g)	19.17 ± 0.13 b	16.75 ± 0.60 a	32.45 ± 1.33 c
H_2O_2 (μmol/g)	14.81 ± 0.57 a	14.87 ± 0.67 a	21.82 ± 0.84 b
O_2^- (nmol/g)	59.29 ± 0.58 a	70.68 ± 1.17 b	154.90 ± 2.13 c
Proline (μg/g)	342.97 ± 8.66 a	355.45 ± 13.01 a	392.15 ± 6.41 b
Chl (mg/g)	2.17 ± 0.01 b	1.82 ± 0.08 a	1.64 ± 0.08 a
Soluble sugar (mg/g)	16.39 ± 0.29 a	24.82 ± 0.87 b	60.50 ± 2.76 c
Soluble protein (mg/g)	8.59 ± 0.21 b	7.46 ± 0.24 a	7.34 ± 0.23 a

Values are mean ± SD (n = 3). CK (non-saline control), SDM (organic fertilizer + microbial inoculant), and SCK (saline-alkali control). Different lowercase letters within a row denote significant differences among treatments (Welch's t-test, $p < 0.05$). SOD, superoxide dismutase; POD, peroxidase; CAT, catalase; MDA, malondialdehyde; H_2O_2, hydrogen peroxide; O_2^-, superoxide anion; Chl, chlorophyll.

Table S3. Grain antioxidant profiles, oxidative stress markers, and quality parameters of wheat

**Indicator**	**CK**	**SDM**	**SCK**
SOD (U/g)	237.76 ± 7.40 a	376.01 ± 15.11 b	447.19 ± 18.91 c
POD (U/g)	125.56 ± 4.93 a	136.42 ± 5.53 a	212.24 ± 6.82 b
CAT (mmol/h/g)	10.04 ± 0.46 a	14.24 ± 0.45 b	16.22 ± 0.30 c
MDA (nmol/g)	4.56 ± 0.21 a	5.18 ± 0.14 b	7.19 ± 0.11 c
H_2O_2 (μmol/g)	1.38 ± 0.06 a	1.59 ± 0.04 b	2.47 ± 0.10 c
O_2^- (nmol/g)	10.56 ± 0.22 a	11.84 ± 0.22 b	12.43 ± 0.31 b
Proline (μg/g)	212.26 ± 1.91 a	230.83 ± 6.33 b	252.00 ± 7.15 c
Total Starch (g/100g)	32.33 ± 1.04 b	30.39 ± 0.54 b	28.21 ± 0.44 a
Crude Protein (g/100g)	8.68 ± 0.26 c	7.86 ± 0.10 b	6.02 ± 0.25 a
Soluble sugars (mg/g)	33.75 ± 1.11 b	32.71 ± 1.25 b	29.28 ± 0.65 a
Soluble protein (mg/g)	7.20 ± 0.07 b	7.34 ± 0.24 b	5.35 ± 0.12 a

Values are mean ± SD (n = 3). CK (non-saline control), SDM (organic fertilizer + microbial inoculant), and SCK (saline-alkali control). Different lowercase letters within a row denote significant differences among treatments (Welch's t-test, $p < 0.05$). SOD, superoxide dismutase; POD, peroxidase; CAT, catalase; MDA, malondialdehyde; H_2O_2, hydrogen peroxide; O_2^-, superoxide anion.

Table S4. Sequencing and rarefaction statistics for 16S rRNA and ITS amplicon libraries.

Sample	Treatment	16S Raw reads	16S OTUs	16S Coverage	ITS Raw reads	ITS OTUs	ITS Coverage
CK1	CK	68498	4195	0.9965	105736	624	0.9995
CK2	CK	69875	4274	0.9967	111908	648	0.9996
CK3	CK	82295	4427	0.9993	112925	657	0.9994
SDM1	SDM	71122	4504	0.9960	108584	700	0.9993
SDM2	SDM	66148	4411	0.9946	102629	652	0.9993
SDM3	SDM	63760	4334	0.9947	114236	709	0.9993
SCK1	SCK	67557	4382	0.9951	102567	541	0.9996
SCK2	SCK	66069	4472	0.9944	104147	736	0.9995
SCK3	SCK	62159	4323	0.9933	99062	626	0.9993

All samples were rarefied to the minimum sequencing depth: 16S = 62159 reads/sample, ITS = 99062 reads/sample. Good's coverage exceeded 0.99 for all libraries.
